# Antihypertensive use insights and experiences among hypertensive patients at Korle-Bu teaching hospital

**DOI:** 10.1371/journal.pone.0298202

**Published:** 2024-06-12

**Authors:** Ezekiel Oti-Boadi, Evans Appiah Osei, Benedicta Asare, Sarah Ampong, Priscilla Ofosuhemaa Asiedu, Fateimah A. Hakami, Paul Akurugu

**Affiliations:** 1 Department of Nursing, Heritage Christian University College, Amasaman-Accra, Ghana; 2 School of Nursing, Purdue University, West Lafayette, Indiana, United States of America; 3 School of Nursing and Midwifery, Valley View University, Adenta-Accra, Ghana; 4 Department of Medical/Surgical, Jazan Health Affairs, Purdue University, Jazan, Kingdom of Saudi Arabia; 5 Special Medicine, Southampton General Hospital, Southampton, Hampshire, United Kingdom; University of Health and Allied Sciences, GHANA

## Abstract

**Background:**

Across the globe, a vast number of people, amounting to 1.28 billion adults aged 30–79 years, suffer from hypertension. Two-thirds of them reside in low- and middle-income countries, and a significant 46% of these adults with hypertension are unaware of their condition. Hence the study aims to determine the Antihypertensive use insights and experiences among hypertensive patients at Korle-Bu Teaching Hospital.

**Methods:**

A qualitative exploratory design was employed to recruit thirty 30 hypertensive patients from both male and female medical wards as well as the hypertensive clinic at the OPD of Korle Bu Teaching Hospital. The patients were selected using a purposive sampling technique, following which they were involved in face-face in-depth interviews which were audiotaped. Recorded data was then transcribed and analyzed with content analysis.

**Findings:**

The analysis of the data resulted in three (3) main themes and 10 sub-themes. The study uncovered a general lack of knowledge about antihypertensive medications. This limited understanding resulted in a negative attitude among most patients toward the use of prescribed antihypertensive drugs. When it came to their experiences with antihypertensive medications, patients shared a wide range of experiences.

**Conclusion:**

It was concluded that there is a need to improve the knowledge and attitude of patients as these play pivotal roles in determining adherence levels. Thus, interventions such as the organization of educational programs and awareness creation is recommended to improve adherence level and in turn, decrease the prevalence of hypertensive complications associated with poor management.

## Introduction

Hypertension emerges as a significant global health issue, impacting a considerable portion of the adult population worldwide. Presently, an estimated 1.28 billion adults aged 30–79 years grapple with hypertension, and the majority, specifically two-thirds, are situated in low- and middle-income countries. An alarming aspect is that 46% of these adults are oblivious to their hypertensive condition [1]. While hypertension is a serious medical concern, it’s noteworthy that effective management, even though without a cure, can play a crucial role in preventing complications and reducing associated risks [2].

Numerous risk factors, including age, gender, physical inactivity, and a high sodium diet, can potentially contribute to hypertension [3]. If left uncontrolled, hypertension may lead to severe conditions such as heart attack, heart failure, stroke, infertility, and kidney failure [4]. Fortunately, there are various anti-hypertensive treatments available globally that aid in managing high blood pressure and mitigating the aforementioned complications, despite the presence of some minor side effects.

Regrettably, the diagnosis and treatment rates for hypertension remain insufficient globally. Less than half of adults with hypertension (42%) receive a diagnosis and appropriate treatment, and only about 1 in 5 adults (21%) have their hypertension under control. This lack of control contributes to hypertension being a significant cause of premature death worldwide [5]. Furthermore, hypertension affects a staggering 26% of the world’s population, equivalent to approximately 972 million individuals. Disturbingly, this prevalence is expected to increase to 29% by 2025, primarily driven by the rising incidence in economically developing nations [6].

Hypertension, including pre-hypertension and other high blood pressure conditions, contributes to a staggering number of deaths worldwide, causing approximately 8.5 million fatalities due to stroke, ischaemic heart disease, vascular diseases, and renal disease [7]. The prevalence of hypertension varies across regions, with the European average reported at 44.2%, whereas in North America, it stands at 27.6% [8]. Within European Union (EU) countries, data from 2019 shows that 22% of individuals aged 15 years and older reported having high blood pressure [9].

Furthermore, there has been a notable shift in the prevalence of hypertension in traditional African societies. Once rare, it has now become a significant public health issue due to the increase in its risk factors [10]. Sub-Saharan Africa bears a considerable burden, with an estimated 74.7 million individuals living with hypertension in the region [8]. In many African countries, including Cameroon, hypertension (HTN) ranks as the most common non-communicable disease [11]. Specifically in Cameroon, surveys report varying prevalence rates of hypertension among individuals above 25 years, ranging from 12% to 22% [12]. Nigeria, with its large population exceeding 170 million, contributes significantly to the overall hypertension burden in Africa [13].

Hypertension poses a significant health challenge in Ghana, similar to other countries. It ranks among the leading causes of hospital admissions and deaths in the country [14]. In 2017, hypertension was the third most common cause of admissions and the leading cause of deaths, accounting for 4.7% of total admissions and 15.3% of total deaths [15]. The outpatient burden of hypertension has been on the rise, particularly in the Greater Accra region. Over a span of five years, the number of new hypertension cases increased by 3.8-fold, from 35,855 in 2006 to 138,040 in 2010 [16]. Hypertension also plays a significant role in medical emergencies such as heart failure and renal failure in Ghana. It is the primary determinant of stroke, with a population-attributable risk of approximately 91% [17]. Against this backdrop, the study aims to investigate the experiences of hypertensive patients regarding the use of antihypertensive medications at Korle-Bu Teaching Hospital.

## Methods

### Research design

Research design refers to the overall strategy utilized to carry out research [18]. An exploratory qualitative approach was utilized to investigate the experiences of hypertensive patients with antihypertensive medication. The study participants included hypertensive patients diagnosed with hypertension for over a year, aged 18 and above, proficient in Asante Twi or English, and willing to participate. Purposive sampling was employed to enhance data trustworthiness and results reliability.

### Sample size

The sample size for the study was determined by data saturation, with this, the researcher considered saturation as the point at which additional data do not lead to any newly emerged themes. Data saturation refers to the stage in the data collection process in research when no new information is discovered [19]. The researcher used 30 hypertensive patients on both Male and Female Medical Wards as well as the hypertensive clinic at the OPD.

The study involved 30 hypertensive patients, with invitations extended to 32 individuals. Two participants declined to participate for personal reasons. The research took place in both the Male and Female Medical Ward as well as the hypertensive clinic at the OPD as these individuals were diagnosed with hypertension and were on antihypertensive medications. As the study progressed to the 27th participant, there was a recurrence of information previously mentioned. Despite the inclusion of three more participants, similar responses continued to be observed. Hence the sample size was obtained on the 30^th^ participant.

### Data collection procedure

Ethical clearance was secured from the Dodowa Health Centre Institutional Review Board (DHC-IRB) which is under Ghana health Service with the protocol identification number DHRCIRB/065/04/22 before the data collection which was done by the first 4 authors. Patients were approached for interviews after explaining the study procedure. Their consent was obtained through a signed form, and meetings were scheduled at their preferred time and place to ensure privacy and confidentiality throughout the process.

Interviews were conducted in person, adhering to COVID-19 protocols, and employing open-ended questions based on the reviewed literature. Male and female patients were purposively sampled. Researchers utilized a semi-structured guide to ensure consistency, recording all data for accuracy. Following the interviews, data was transcribed and analyzed. Each interview, lasting 1–2 hours, comprehensively explored the topics. Data collection spanned 3 months (June to August 2022).

This study primarily employed a semi-structured interview guide designed to align with the study’s objectives. The guide featured open-ended questions and probing prompts for in-depth exploration. After obtaining approval from the Dodowa Health Research Centre Institutional Review Board (DHRCIRB/065/04/22), a pilot study involving five Ridge Hospital patients was conducted to clarify any uncertainties before the main interviews. Insights from the pilot study significantly contributed to the refinement of the semi-structured interview guide, and Ridge Hospital was chosen as the pilot site due to its similarity in managing hypertension patients to the main study site.

### Data analysis

Data analysis involves organizing information into manageable themes, patterns, trends, and relationships [20]. Content analysis, on the other hand, is the process of identifying specific words, themes, or concepts within qualitative data [21]. As described by [22], content analysis comprises familiarization, coding, categorization, and theme development. The themes were organized using NVivo 12 software.

During familiarization, the researcher engaged with the data by reading and re-reading interview transcripts to understand prevalent topics discussed by patients. Coding involved identifying words or sentences with similar meanings [23]. Categorization grouped related codes within their context, while theme development expressed the underlying meaning by organizing data into themes and subthemes [24]. Data Analysis was done by all the authors.

## Methodological rigour

Methodological rigor, as outlined by [25], is integral to establishing confidence in the conclusions drawn from evaluation results. The key components of this rigor—credibility, transferability, dependability, and confirmability—play crucial roles in ensuring the trustworthiness and reliability of the study. Credibility was upheld through practices such as prolonged engagement during data collection (spanning 3 months) and conducting in-depth interviews (each lasting 2 hours), coupled with member checking for manuscript review by researchers before publication. Transferability was addressed by providing comprehensive descriptions of the methods, including details about the population and sample size. Dependability was achieved through transparent documentation, emphasizing the stability and consistency of findings over time. Confirmability, focusing on objectivity, was maintained through techniques like reflexivity, where verbatim quotes are utilized to faithfully reflect participants’ views, preventing the influence of researchers’ personal views and biases on the results.

## Results

### Analysis of socio-demographic data

The study was done using 30 respondents receiving treatment for hypertension. The results revealed that 18 out of the 30 participants (60.0%) were female, while the remaining 40% were male. The age of participants ranged from 31 to 55 years, with 36.7% falling between 51 to 55 years. In terms of educational status, only 16.7% had attained a tertiary level of education, and the majority (80.0%) identified as Christians. Additional details are provided in Table 1.

**Table 1:** Socio-demographic characteristics of participants.

Variable	Frequency(n = 30)	Percentage (%)
**Age group**		
30–35	5	16.7%
36–41	10	33.3%
42–47	4	13.3%
48 and above	11	36.6%
**Gender**		
Male	12	40.0%
Female	18	60.0%
**Denomination**		
Christian	13	43.3%
Muslim	17	56.7%
**Employment Status**		
Employed	30	100.00%
**Marital status**		
Single	8	26.7%
Married	19	63.3%
Divorced	2	6.7%
Separated	1	3.3%
**Educational Status**		
Primary	13	43.3%
Secondary	12	40.0%
Tertiary	5	16.7%
**Languages spoken**		
Ga	7	23.3%
Ewe	9	30.0%
Hausa	3	10.0%
Akan	11	36.7%
Total	30	100%

### Organization of the themes

There were 3 main themes and 10 sub-themes**. See Table 2 for further details.**

**Table 2:** Themes and subthemes.

THEMES	SUBTHEMES
1. The knowledge level of patients on antihypertensive medications.	• Storage and dosages of antihypertensive medications. • Side effects of antihypertensive medications. • Drug–drug interaction Frequency and duration
2. The attitude of patients towards the use of antihypertensive medications 3. Patient’s accounts of inconsistencies in antihypertensive usage	• Concerns about sexual performance • Combining orthodox with herbal medicine • Antihypertensive non adherence • Issues with forgetfulness • Financial challenges • Anti-hypertensive medication taste concerns • Family support for antihypertensive medication adherence

### Antihypertensive medications knowledge

#### Storage and dosages of antihypertensive medications

There was a noticeable variation in the patients’ knowledge regarding the storage and dosage of antihypertensive medications. It became evident that a significant proportion of the study participants lacked sufficient knowledge concerning the appropriate dosage of antihypertensive medications. The responses provided below further reinforced this observation:

*“Erhmmmm…*.*with regards to the storage I really don’t know whether there is any special place to keep the medication but I do keep it anywhere*. *I remember one time*, *I placed it close to my cooker in the kitchen because whilst I was cooking I started having headache and I knew my blood pressure was high*, *so I quickly instructed my daughter to bring it and after taking it I forgot it there”* P26

“*Well for me I normally keep it in my cupboard where I keep important documents that I use for work because that place is high and far from where my kids can reach*, *you know how these kids can be like always running and playing with anything and everything*. *So yeah I would say my storage place for my medication is my cupboard”* P15

Meanwhile, a few of the patients were aware of the appropriate way of storing their antihypertensive medications in order to retain its potency and efficacy.

*“I know that generally*, *medications are to be stored in a cool and dry place*, *not close to any form of heat or directly under sunlight because these harsh conditions can change the chemical component of the medication and make it poisonous to the body*. *So I always make sure that my medication is in my a cool area because I don’t want to be taking toxins into my body all in the name of medication”***P3**

Some of the patients indicated that they were not knowledgeable about the dosage of the antihypertensive drugs they were using and therefor had to consult their health professionals. One of the patients posited that:

“*Well*, *for me I know I am supposed to take only one of the tablet each*, *but I really don’t know the milligram*, *so one time when I ran out of the medication and I had to buy from a pharmacy they didn’t have the brand of the medication I normally used but they had another brand; however*, *because I didn’t know the milligram I had to wait and confirm from my doctor*.*”* **P8**

#### Side effects of antihypertensive medications

The study findings also shed light on the patients level of knowledge regarding the side effects associated with different antihypertensive medications used in managing their condition. It was observed that the patients had some degree of knowledge in this area, primarily based on their personal experiences with drug usage. The following comments from patients reflect their perspectives on this matter:

“*I know of frequent urination*, *this is because one of the medication named lasix makes me urinate very frequently*. *Whenever I take it you will notice that I am going to the bathroom more frequently than I used to*. *Another side effect is dizziness; one time I almost fell so in one of my reviews*, *I told the nurses about it but they told me it was a side effect and that if I should experience it more frequently I should report but ever since then it did not occur”*P30

*“For me*, *whenever I use the medication*, *I feel so weak and fatigued that I can hardly do anything useful “*P1

### Drug-drug interaction, frequency and duration

The study revealed a significant lack of knowledge among the patients concerning drug-drug interactions. On the other hand, it was evident that the majority of patients had a good understanding of the frequency with which they should take their medication. This observation is exemplified in the following verbatim quote:

“*Me I don’t know of any medication that could react with taking my hypertensive drugs; I usually take some of my herbal preparations along with the medications because I heard the herbal one works very well*, *so most times I combine the herbal medications with the orthodox*, *so that I can take a maximum effect”***P11**

a few denoted that they were aware of the concept; however, the knowledge on the medications not to be taken together with their antihypertensive drug was lacking. They indicated that:

“*Hmmmm…*..*I have heard my elder brother who is a doctor say something like that*, *so I am very much aware of the fact that some medications cannot be combined but I don’t know of any medications that I shouldn’t take together with my hypertensive medications”***P20**

*“I know that the use of over-the-counter medications with hypertensive medications are not good*, *so whenever I have any health issue*, *I make sure I go to my doctor as he is in the best position to determine which medications I could use which will not react with the hypertensive medication I am already taking”* **P14**

### The attitude of patients towards the use of antihypertensive medications

#### Concerns about sexual performance

Some of the patients were seen to be worried about the impact of the use of antihypertensive medications on their sexual performance; a finding that was more common amongst the males. This concern was depicted by the following responses:

“*Hmmmmm……*.*this hypertensive drugs I think is affecting my sexual life because ever since I started taking it I feel like my sex drive has reduced and this is really bothersome to me because I have a young wife as an Alhaji who I married somewhere last 2 years…*.*i am so worried about it”***P29**

*“I am very concerned about my sexual performance due to this medication because I read online that some hypertensive medications can affect sexual libido and I don’t want that to happen because I am a young woman and my spouse is also young”* **P1**

#### Combining orthodox with herbal medicine

The study findings revealed that whilst some individuals still made use of the orthodox medications others combine both as the combination of both was perceived to be more effective as compared to just using orthodox. The following responses affirmed this fact:

“*I take some of my herbal preparations along with the medications because I heard the herbal one works very well*. *I was even told that the herbal one has cured a lot of people from this hypertension*, *so I am combining both so that it will work as fast as possible and I can be cured completely because taking these medications almost every day is not an easy task”*.**P17**

“*Yes*, *I believe in the herbal preparations; so whenever my mother is coming to town I make her get some herbal preparation for my hypertension for me*. *I know herbal medications even works faster than the ones given at the hospital but this does not mean I do not take the ones prescribed here at the hospital; I do take them both and as a matter of fact I use the liquid herbal preparation for swallowing my hypertensive drugs most of the time”* **P19**

Whilst some took both herbal and orthodox medications simultaneously, others took them at different interval. A few of the patients stated that:

“*Well…like I said earlier*, *I normally combine both the herbal preparation given to me by a well-known Muslim herbalist at Kasoa with the drugs given to me at the hospital*. *So what I do is that I take the hospital medications and then the herbal preparation the subsequent day*, *so that I will have maximum effect”* **P6**

### Patient’s accounts of inconsistencies in antihypertensive usage

#### Antihypertensive non-adherance

In light of the study findings more than half of the patients were found to be inconsistent in terms of taking their medications as prescribed and this could have adverse impact on their condition; nonetheless, most of them may not be aware of the adverse effect this attitude holds thus the reason for their actions. The patient’s responses are as follows:

“*I don’t usually take the medications as prescribed and that is the main reason why I have been admitted here because I had not taken it for about two weeks and whilst I was at a meeting I just fell and passed out and the next thing is that I woke up finding myself in this hospital with my family members all around me*. *They said my blood pressure went too high and that was why I fainted”* **P30**

“*You know as a market woman there are lots of things I have to do every day*, *I mean I have to supply eggs and other stuffs so most time there is no opportunity to even take the medications*, *so I don’t really take it as regular as the doctor has prescribed it for me…”***P15**

Some of the patients also indicated that their inconsistency was merely as a result of being tired of constantly taking medications every day for a condition that never goes away. They verbalized that:

“*For me it’s not like I forget or anything of that sort; I am just tired of taking these medications that sometimes I just choose to skip taking it and just relax for the day*. *I mean I have been on these medications for over ten years and sometimes it can be tiring…it is not easy …to take them frequently”***P26**

#### Issues with forgetfulness

Another factor that was highlighted by most of the patients which contributed to poor adherence to antihypertensive medications was the habit of forgetting to take medications. The following statements affirmed this fact:

“*As an accountant I have a very busy schedule such that I forget to take my hypertensive medication and even sometimes I forget to eat*, *so I would say that is one reason why I am unable to fully adhere to taking the hypertensive drugs as prescribed*. *Even on days when I am on leave or off duty*, *I still forget because I am always engaged with an activity”* **P3**

“*It is not easy at all oh*, *being an Alhaji*, *having a business and a big family to take care of can be so stressful sometimes that I forget to even eat talk of taking the medications*. *That is the reason why I am admitted now because for the past one week I had some family issues to deal with and I totally forgot I was hypertensive and was not taking my drugs now see where it has landed me into”***P11**

“*I normally forget most times especially when my daughter is not around sometimes I do forget for a whole week and when I begin to get the headaches I then remember I have to take the medications”***P23**

#### Anti-hypertensive medications taste concerns

The taste of the drug was another factor that was identified to make the experience of some of the patients a little bit uncomfortable; thus making it difficult to adhere to the treatment regimen. The comments are as follows:

“*The main reason why I don’t take the drugs regularly is due to the taste of one of the medication*. *I think the name is captopril; every time I take this drug my mouth becomes bitter all day that I cannot even enjoy any food*, *it’s just so annoying and as a result of this I don’t take it as prescribed”***P13**

“*Yes the taste of the drugs is not good kraaa…I mostly feel noxious every time I take it so for me I don’t like it and I am wondering if I could get a change of the medication because I was just diagnosed 3 months ago and if I have to take this drug the rest of my life then I need something that my system will be more receptive of”***P10**

### Financial challenges

The responses of the patients made it evident that some face difficulties adhering to prescribed treatment regimen due to financial problems. The comments are as follows:

“*Some of the hypertensive medications prescribed for me has become very expensive and with the prices of everything becoming very high I am unable to purchase it”* **P20**

“I *am a trader who earns very little from my business; using the money earned to cater for myself and my family is almost impossible as the money is incapable of providing food for the family*. *For this reason*, *I am unable to afford the hypertensive medications most times”***P8**

“*Buying the medicine is one problem*, *every two or one week I have to be buying these medications*. *I don’t make that much from my shoe business and aside that too I have to cater for my entire family*. *So the money to even spend on these drugs is not there”***P24**

However, a few patients did not experience the challenges in terms of finances. They stated that:

“*As for me…*.*I won’t say financial challenges is a reason why I do not correctly adhere to taking my hypertensive drugs because I have the money and I always buy them in bulk”***P15**

### Family support for antihypertensive medication adherence

The support provided by family members helps to improve patients’ health and sense of well-being which in turn could increase their adherence to prescribed treatment regimen. As per the responses of patients, support came from parents, spouses and even children:

“*Throughout this journey*, *it has not been easy; I mean having to take these medications and maintain a certain lifestyle to make sure my blood pressure is in check has been difficult but I thank God that my wife has been very supportive*. *She is the one who always reminds me about taking my drugs and she also makes sure I am eating more fruits and vegetable with less cholesterol*, *in fact I can say her support has helped me*, *like by now I would have died of very high blood pressure if not for her consistent support “*P6

“*Hmmm…*..*my whole family members has been supporting me in different ways ever since I was diagnosed with hypertension; my children make sure my medication is always ready and available and that I get regular checks and providing the money needed*. *Additionally*, *my husband and I are retirees and we are always home together with no business or kids to take care of; so he makes sure I am checking my blood pressure as well as taking the medication*. *The support has been amazing and I must say it has really been helpful”*P25.

Whilst most of the patients indicated that they received support from their families; a few also highlighted that their families have not been supportive from day one:

“*My family has never really supported me; I mean even though they know I have hypertension now none of them really shows concern*. *Nobody makes an effort to visit me or even take the time to check on me through calls or messages to see how I am coping with the hypertension*. *No one really care about me and this is so heartbreaking sometimes”* P17

## Discussions

The study found that a majority of participants lacked knowledge about their antihypertensive medication dosages, with only a few being able to state the correct dosage. In contrast, a study by [26] reported that their participants had sufficient knowledge about antihypertensive medication use, positively influencing blood pressure control. Socio-demographic variations among patients could explain the disparity in findings [27]. Despite this, the current study highlights the need for consistent patient education during hospital visits to improve knowledge on appropriate drug dosage.

Participants in the study were aware of antihypertensive medication side effects, including frequent urination, dizziness, fatigue, and nausea, gained through personal experiences, healthcare providers, or the internet. [28] similarly reported good knowledge of side effects. Despite variations in reported side effects between studies, consistent education efforts are crucial. Sufficient knowledge alone may not ensure adherence, as highlighted by [29], emphasizing the need to dispel misconceptions and include strategies for managing side effects, stressing the importance of consulting healthcare providers for adverse effects.

The patients’ knowledge regarding drug-drug interactions was found to be deficient in this current study, as more than half of them indicated their lack of awareness regarding any potential interactions with their antihypertensive medications, and some were unfamiliar with the term drug-drug interaction. This discovery is worrying as hypertensive patients are typically prescribed antihypertensive medications for a lifetime in addition to other medications, making the potential interactions between antihypertensive agents and other drugs a significant concern. Conversely, patients’ lack of awareness regarding drug-drug interactions may elevate the risk of such interactions occurring, as supported by a study that found a positive correlation between the presence of illnesses, co-morbidities, and poly-pharmacy, and the increased incidence of drug-drug interactions in hypertensive patients [30–33].

Participants expressed concerns about their sexual performance, particularly regarding the potential impact of certain drugs on libido. These concerns were more pronounced among males, as some antihypertensive medications have been linked to issues like erectile dysfunction. However, it’s worth noting that no single class of antihypertensive medication has been associated with sexual dysfunction in women, as evidenced by [34]. To address these concerns and prevent potential poor adherence and complications, appropriate interventions should be implemented [35].

A majority of the patients expressed various reasons for their inconsistent adherence to their prescribed antihypertensive medication. Some cited their busy schedules and the demanding nature of their work as hindrances to following the treatment schedule, while others simply grew weary of the daily medication routine. This aligns with a study conducted by [36], who revealed that patients exhibited inconsistency in taking antihypertensive medications, with only approximately 32.1% of them being observed to adhere closely to the prescribed treatment regimen. Some reasons for non-compliance included lack of knowledge, ignorance of the need for long term treatment, religious practices and cultural beliefs [36]. Interestingly, [37] found that more than half of the study participants had positive attitude towards antihypertensive medications.

The study revealed diverse experiences among patients with antihypertensive medication usage. For over half of them, financial constraints hindered medication adherence, in line with [38] study in Ghana. These parallel findings emphasize the pressing need to enhance antihypertensive medication affordability in Ghana, where hypertension is a significant public health issue [39]. Forgetfulness, often due to busy schedules, affected adherence. A study in Samarkand, Uzbekistan, found few patients citing forgetfulness as a reason [40], while other studies [41, 42] reported forgetfulness as a common contributor to suboptimal adherence. This underscores the importance of enhancing patient’s knowledge to improve adherence.

While various factors influenced low adherence to antihypertensive medication, family support notably encouraged patient’s medication intake. Those receiving support from family members reported a better experience. A study in Nigeria by [43] similarly linked family support to improved blood pressure control and treatment adherence. Family support is crucial in managing various health conditions, with numerous studies affirming its potential for enhancing adherence and population-level blood pressure control [44–46]. Therefore, healthcare providers should encourage family support for blood pressure management. In general, patients experiences with antihypertensive medication varied due to diverse factors.

## Conclusions

In conclusion, this study exposes knowledge gaps in participants regarding antihypertensive medication dosages, emphasizing the crucial role of consistent patient education during hospital visits. Despite awareness of side effects, obtained through personal experiences and healthcare providers, ongoing efforts are needed for comprehensive knowledge. The study also reveals a concerning lack of awareness about drug-drug interactions among hypertensive patients, highlighting the need for increased education in this area.
